# Excision of Integrated Human Herpesvirus 6A Genomes Using CRISPR/Cas9 Technology

**DOI:** 10.1128/spectrum.00764-23

**Published:** 2023-03-16

**Authors:** Giulia Aimola, Darren J. Wight, Louis Flamand, Benedikt B. Kaufer

**Affiliations:** a Institut für Virologie, Freie Universität Berlin, Berlin, Germany; b Division of Infectious and Immune Diseases, CHU de Quebec Research Center-Laval University, Québec, Canada; c Department of Microbiology, Infectious Disease and Immunology, Faculty of Medicine, Laval University, Québec, Canada; d Veterinary Centre for Resistance Research (TZR), Freie Universität Berlin, Berlin, Germany; University of Wisconsin-Madison

**Keywords:** HHV-6, iciHHV-6, integration, CRISPR/Cas9, gRNAs, excision

## Abstract

Human herpesviruses 6A and 6B are betaherpesviruses that can integrate their genomes into the telomeres of latently infected cells. Integration can also occur in germ cells, resulting in individuals who harbor the integrated virus in every cell of their body and can pass it on to their offspring. This condition is termed inherited chromosomally integrated HHV-6 (iciHHV-6) and affects about 1% of the human population. The integrated HHV-6A/B genome can reactivate in iciHHV-6 patients and in rare cases can also cause severe diseases including encephalitis and graft-versus-host disease. Until now, it has remained impossible to prevent virus reactivation or remove the integrated virus genome. Therefore, we developed a system that allows the removal of HHV-6A from the host telomeres using the CRISPR/Cas9 system. We used specific guide RNAs (gRNAs) targeting the direct repeat region at the ends of the viral genome to remove the virus from latently infected cells generated *in vitro* and iciHHV-6A patient cells. Fluorescence-activated cell sorting (FACS), quantitative PCR (qPCR), and fluorescence *in situ* hybridization (FISH) analyses revealed that the virus genome was efficiently excised and lost in most cells. Efficient excision was achieved with both constitutive and transient expression of Cas9. In addition, reverse transcription-qPCR (RT-qPCR) revealed that the virus genome did not reactivate upon excision. Taken together, our data show that our CRISPR/Cas9 approach allows efficient removal of the integrated virus genome from host telomeres.

**IMPORTANCE** Human herpesvirus 6 (HHV-6) infects almost all humans and integrates into the telomeres of latently infected cells to persist in the host for life. In addition, HHV-6 can also integrate into the telomeres of germ cells, which results in about 80 million individuals worldwide who carry the virus in every cell of their body and can pass it on to their offspring. In this study, we develop the first system that allows excision of the integrated HHV-6 genome from host telomeres using CRISPR/Cas9 technology. Our data revealed that the integrated HHV-6 genome can be efficiently removed from the telomeres of latently infected cells and cells of patients harboring the virus in their germ line. Virus removal could be achieved with both stable and transient Cas9 expression, without inducing viral reactivation.

## INTRODUCTION

Human herpesvirus 6A and 6B (HHV-6A/B) are closely related betaherpesviruses (1–4). Primary infection with HHV-6B usually occurs within the first 2 years of life. It is the causative agent of the febrile illness roseola infantum (5), which can be accompanied by more severe neurological complications like seizures and encephalitis. HHV-6A infection is believed to occur later in life, but less is known about its epidemiology and clinical manifestations (5–7). Upon primary infection, HHV-6A/B can establish latency, allowing the virus to persist in the host for life (8, 9). While most herpesviruses maintain their genome as a circular episome during latency, HHV-6A/B can integrate into the telomeres to maintain their genome (10–12). Intriguingly, integration can occur not only in somatic cells but also in germ cells. Integration in germ cells can result in individuals carrying the integrated virus in every cell of the body, and they can pass on the integrated virus to their offspring in a Mendelian fashion (10, 13–15). This condition is known as inherited chromosomally integrated HHV-6 (iciHHV-6) and affects about 1% of the world population. HHV-6A/B reactivation in iciHHV-6 individuals and from its latent state has been associated with different clinical manifestations, including heart diseases, encephalitis, and graft-versus-host disease (GvHD) (16–21).

HHV-6A/B have a linear double-stranded DNA genome of approximately 160 kbp. The unique region (U) contains most viral genes and is flanked by two identical direct repeat regions (DRs) of about 8 kbp each (4, 22–24). The DRs also contain telomeric repeat arrays (TMRs) consisting of (TTAGGG)*_n_* repeats identical to the telomeres of humans and all other vertebrates (25–28). These viral TMRs have been previously shown to facilitate the integration of HHV-6A into the host telomeres (29).

Analyses of the integrated virus in iciHHV-6 patients revealed that integration occurs with a specific orientation of the virus genome (30–32). The perfect TMR array (pTMR) on the right DR apparently recombined with the host telomeres. Thereby, longer telomere sequences of several kilobase pairs remain between the virus and the subtelomeres of the host chromosome. At the end of the left DR, long telomeres were observed that correspond to the length of the other telomeres within the host cell (33). Several viral and cellular factors have been investigated (29, 34–39); however, the precise mechanism that facilitates HHV-6 integration remains poorly understood. Until now, it has remained impossible to prevent HHV-6A/B reactivation and/or eliminate the integrated virus from human chromosomes, which we aimed to achieve in this study.

The clustered regularly interspaced short palindromic repeats (CRISPR) and the CRISPR-associated (Cas) protein system are an adaptive immune mechanism in prokaryotes that targets and destroys the nucleic acids of invading viruses (40–43). Among the different CRISPR/Cas classes and groups, the CRISPR type II endonuclease Cas9 of Streptococcus pyogenes is one of the best-studied tools (44, 45). This CRISPR/Cas9 system consists of two main components that act together as a complex: the Cas9 endonuclease and a specific guide RNA (gRNA) that drives Cas9 to the desired target sequence (46–49). In the last several years, the CRISPR/Cas9 system has revolutionized the field of genome editing and proved to be a powerful tool for many applications (45–47, 50–53).

In this study, we established a system that allows removal of the integrated HHV-6A genome from the host telomeres using the CRISPR/Cas9 technology. Elimination of the virus genome was achieved in latently infected cells generated *in vitro* and iciHHV-6A patient cells. Both constitutive and transient expression of Cas9 facilitated efficient removal of the virus genome. Successful excision was validated by fluorescence-activated cell sorting (FACS), quantitative PCR (qPCR), and fluorescence *in situ* hybridization (FISH), and the absence of viral reactivation upon excision was assessed by reverse transcription-qPCR (RT-qPCR). Our study provides the first evidence that the HHV-6A genome can be efficiently removed from the host telomeres, an approach that could be used to investigate important features of HHV-6 biology and potential clinical applications in the future.

## RESULTS

### Generation and characterization of Cas9-expressing cells for the removal of the HHV-6A genome.

To establish the removal of the integrated HHV-6A genome with the CRISPR/Cas9 system, we first used a well-characterized 293T cell line harboring the integrated virus genome (293T-6A) (54, 55). These cells were previously infected with HHV-6A expressing green fluorescent protein (GFP) under the control of the major immediate early human cytomegalovirus (HCMV) promoter and harbor two copies of the integrated virus genome. Cas9 was delivered into 293T-6A cells by lentivirus transduction to generate a polyclonal population stably expressing Cas9 upon puromycin selection (293T-6A-Cas9).

**FIG 1:**
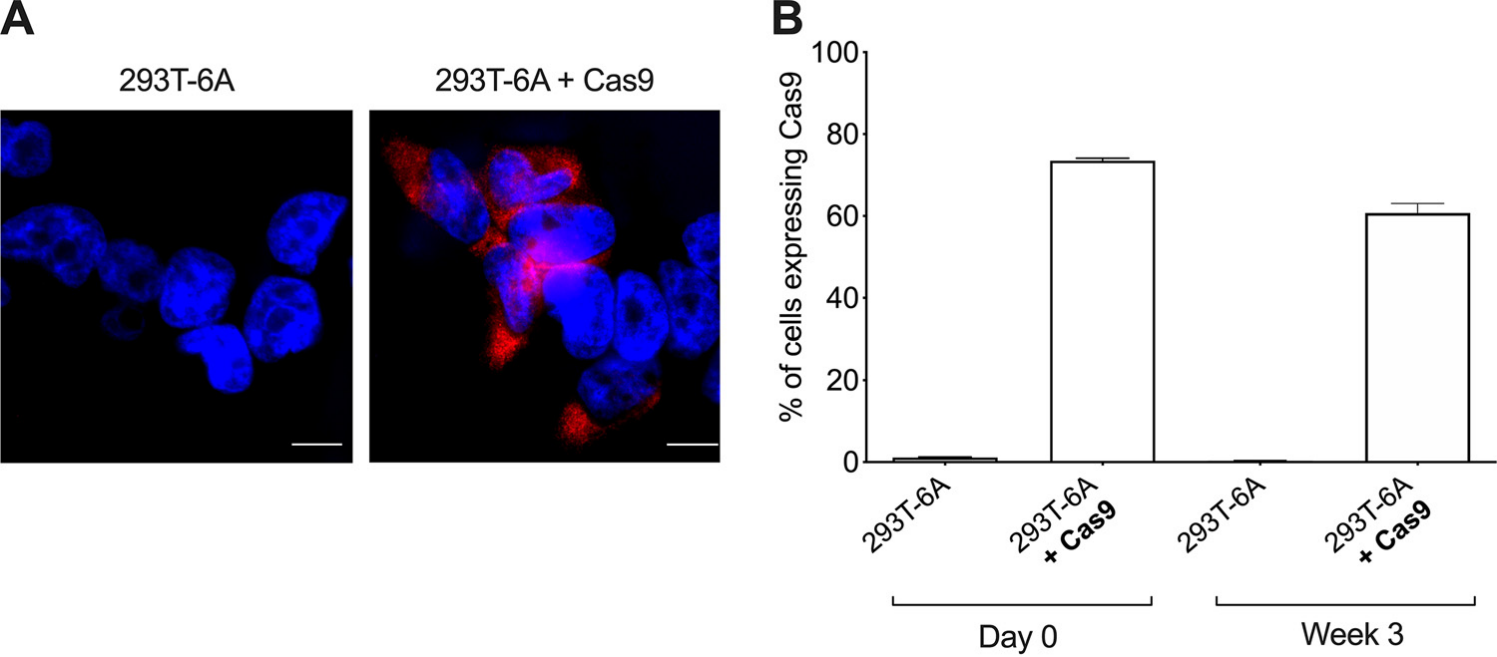
Quantification of Cas9 expression in transduced 293T-6A cells. (A) Cas9 expression was assessed by immunofluorescence using an anti-d-Tag antibody and a secondary Alexa Fluor 568-conjugated antibody (red). Cell nuclei were stained with 4′,6-diamidino-2-phenylindole (DAPI) (blue). Untransduced 293T-6A cells were used as a negative control (bar, 10 μm). (B) Quantification of Cas9 expression was performed by flow cytometry (FACS). Results are shown as means from three independent quantification (one-way ANOVA; *n* = 3; ±standard error of the mean). Representative FACS plots with gating are shown in Fig. S1 in the supplemental material.

Prior to the delivery of HHV-6A specific gRNAs (6A-gRNAs), we assessed the Cas9 expression in the population. Immunofluorescence staining revealed that not all cells expressed the Cas9 protein despite the selection (Fig. 1A). We therefore decided to quantify the percentage of Cas9-positive cells by flow cytometry. The data revealed that about 70% of the cells expressed Cas9 at various levels. The remaining 30% appeared to be Cas9 negative, suggesting that Cas9 is either quickly downregulated or not expressed upon lentivirus delivery (Fig. 1B, day 0, and see Fig. S1 in the supplemental material). Moreover, the percentage of Cas9-expressing 293T-6A cells decreased over time, indicating that there is a selection pressure against Cas9 expression (Fig. 1B, week 3). Similarly, when we attempted to make Cas9-expressing clones, the production of the protein decreased over time. By the time enough clonal cells were available for the experiments, the percentage of Cas9-positive cells was comparable to that of the original polyclonal population. Therefore, we decided to perform the initial experiments with the polyclonal population.

### Removal of integrated HHV-6A genomes using specific gRNAs.

**FIG 2:**
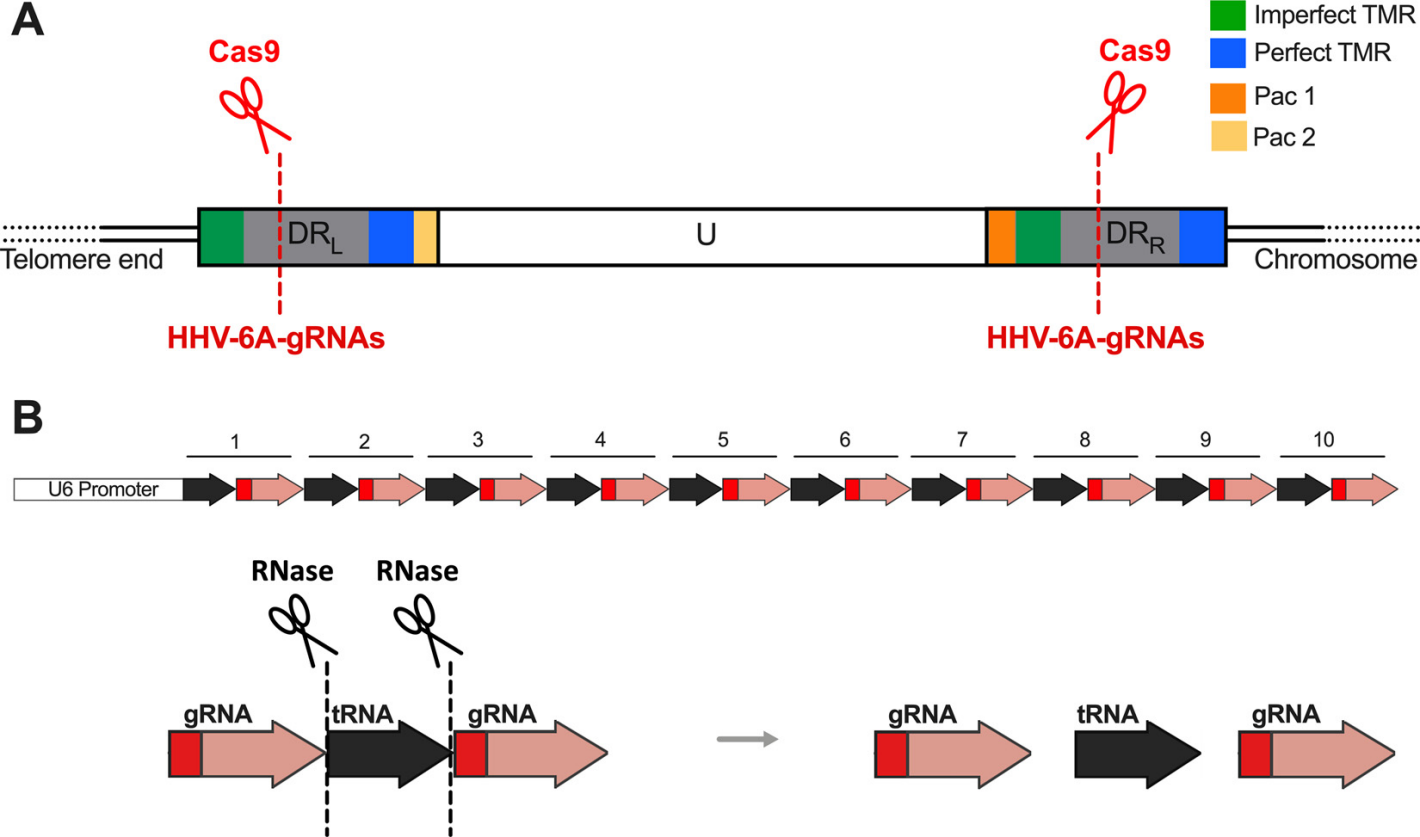
Schematic representation of HHV-6A genome and polycistronic-tRNA-gRNA (PTG) construct. (A) The specific HHV-6A gRNAs designed (red dashed line) were all directed against the noncoding region of the DRs (gray boxes). Driven by the gRNAs, Cas9 protein will excise the HHV-6A genome from the host telomeres. (B) In the PTG construct, the specific HHV-6 gRNAs (red arrows) are interspersed with tRNA sequences (black arrows) and transcribed as a single polycistronic RNA from the U6 promoter. Following transcription, the endogenous cellular RNases will precisely cut out the tRNA sequences, allowing the release of the individual HHV-6A-specific gRNAs.

To excise the HHV-6A genome, we delivered the second component of the CRISPR/Cas9 system, HHV-6A-specific gRNAs (6A-gRNAs). We designed 10 gRNAs directed against the noncoding region of the DRs at the end of the viral genome (Fig. 2A and Table 1). To deliver multiple gRNAs at once, we used the polycistronic-tRNA-gRNA (PTG) system (56, 57). In this system, the individual gRNAs are interspaced by tRNA sequences and driven by a single U6 promoter (Fig. 2B). Upon transcription, tRNAs are removed by endogenous RNases, resulting in the release of individual gRNAs (Fig. 2B). As a control, we delivered gRNAs targeting another herpesvirus (Marek’s disease virus [MDV]) established previously (58).

**TABLE 1:** HHV-6A-specific gRNA sequences (only target sequence shown)

gRNA	Target sequence (5′-3′)	PAM^*a*^ (3′)	Orientation
gRNA_1	AGGGGCCGGACGTATACGAA	GGG	Antisense
gRNA_2	CTCGTTGCGAAACCGCAACA	GGG	Sense
gRNA_3	AGTCCGTAAAATCACCCGAG	GGG	Sense
gRNA_4	GAAACCGAAAGCGTAAAGGG	CGG	Sense
gRNA_5	CTGTCGCTGTTCGGAGAACG	TGG	Antisense
gRNA_6	GGAAAGGGGACGTACGGAGA	AGG	Antisense
gRNA_7	GTCGGACCTCGGGTCCGAAG	CGG	Antisense
gRNA_8	CGCCAGACATCGACCGCCGG	AGG	Sense
gRNA_9	GTTGCCGTAGAGGCGTCCGA	GGG	Antisense
gRNA_10	TACCGAATGCAAAAAGTTAA	AGG	Sense

a PAM, protospacer-adjacent motif.

**FIG 3:**
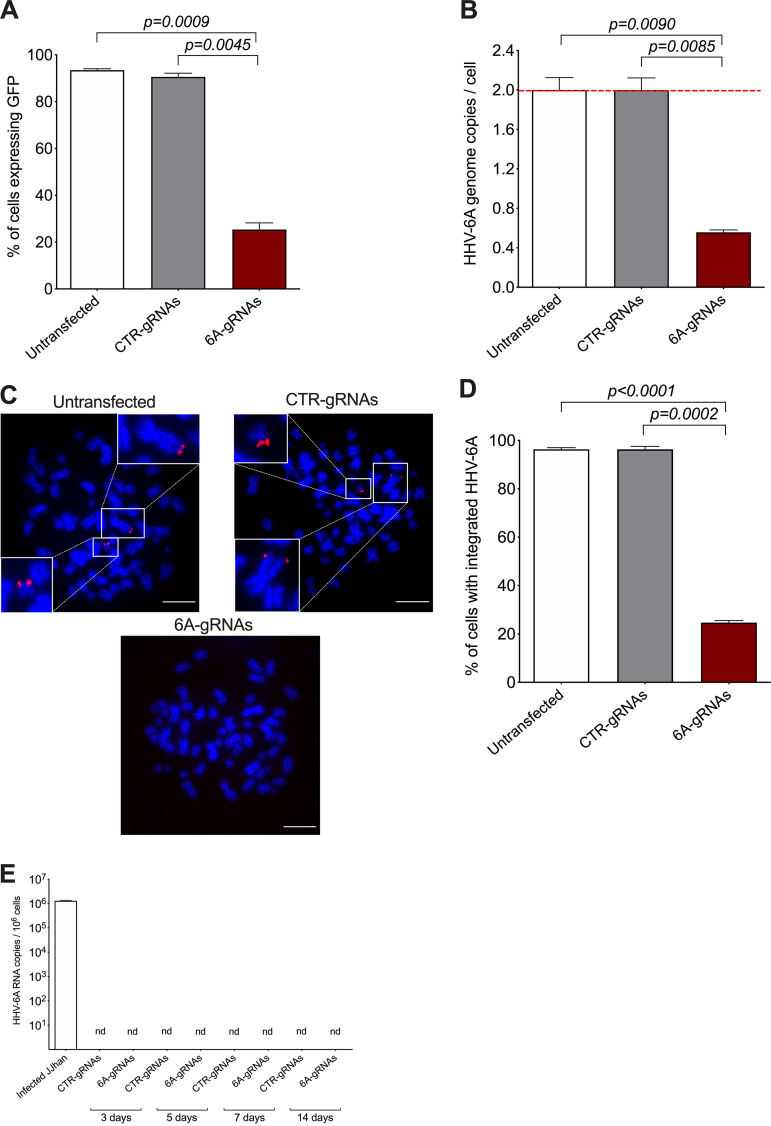
Removal of integrated HHV-6A genome in Cas9-transduced 293T-6A cells. (A) Virus-encoded GFP was detected after TPA stimulation by flow cytometry in untransfected 293T-6A-Cas9 cells or cells transfected with control (CTR) gRNAs or HHV-6A-specific gRNAs. Results are shown as means from three independent experiments (one-way ANOVA; *n* = 3; ±standard error of the mean). Representative FACS plots including gating strategy are shown in Fig. S2 in the supplemental material. (B) Copy numbers of the HHV-6A U86 gene were detected by qPCR. Results are shown as fold change compared to untransfected 293T-6A-Cas9 cells (red dashed line), which harbor two copies of the integrated virus (one-way ANOVA; *n* = 3; ±standard error of the mean). (C) Integrated virus was detected by FISH using specific HHV-6A biotin-labeled probes and Cy3-streptavidin antibody (red). Metaphase chromosomes were stained with DAPI (blue). Representative images of indicated conditions are shown in untransfected 293T-6A-Cas9 cells, cells transfected with control gRNAs, and cells transfected with specific HHV-6 gRNAs (bar, 10 μm). (D) One hundred interphase nuclei were examined for the presence or absence of the integrated virus (one-way ANOVA; *n* = 3; ±standard error of the mean). (E) Total RNA was extracted at indicated time points after gRNA transfection. The absence of viral transcripts was confirmed for the HHV-6 immediate early gene U86 (*n* = 3; ±standard error of the mean) by qPCR. As a positive control, RNAs from lytic infected JJhan cells were analyzed. nd, not detected.

**FIG 4:**
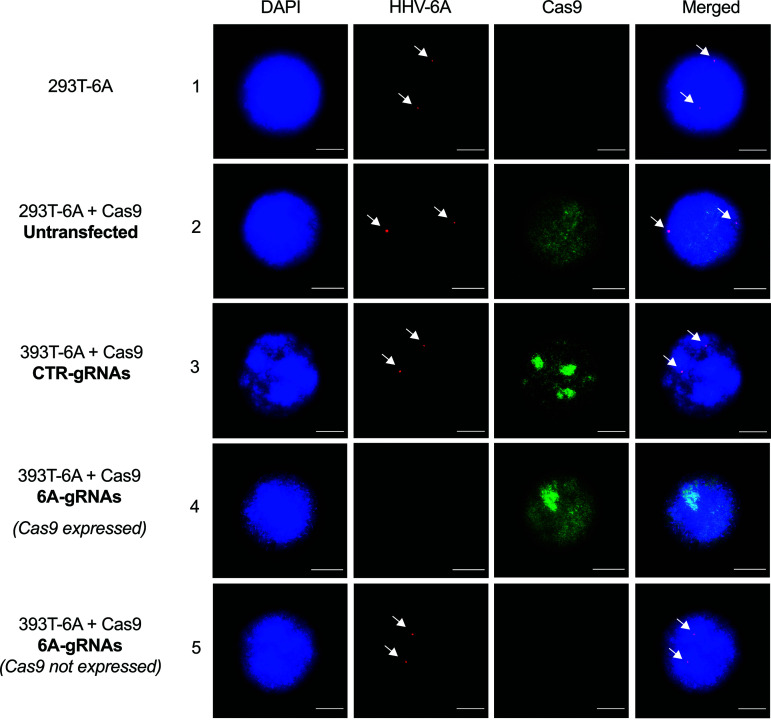
Fluorescence *in situ* hybridization to simultaneously detect integrated HHV-6 and Cas9 protein. Integrated virus was detected by FISH using specific HHV-6A biotin-labeled probes and Cy3-streptavidin antibodies (red). Metaphase chromosomes were stained with DAPI (blue). Cas9 protein was detected with an anti-Cas9 Alexa Fluor 647-conjugated antibody. The arrows indicate the integrated virus genome. Representative images are shown (bar, 10 μm).

After transfection of the 293T-6A-Cas9 cells with the 6A-gRNA expression plasmid, we selected the cells using hygromycin and validated the excision by three independent methods. First, we made use of the GFP reporter in the virus genome that is expressed in the cells upon stimulation with phorbol 12-myristate 13-acetate (TPA). FACS analyses revealed a reduction in GFP-expressing cells of about 70% using the 6A-gRNAs, indicating that the HHV-6A genome was successfully removed in most cells (Fig. 3A and Fig. S2). In addition, we assessed the removal of the virus genome by qPCR and observed that the HHV-6A genome copies were also reduced by about 70% compared to the controls (Fig. 3B). Finally, we performed fluorescence *in situ* hybridization (FISH) to visualize the virus genome. In the untransfected and control gRNA-transfected cell, two copies of the integrated virus were readily detected at the ends of metaphase chromosomes using an HHV-6A-specific probe (Fig. 3C, upper images). In contrast, the integrated virus was no longer detectable in most cells transfected with the 6A-gRNAs (Fig. 3C, lower image). We quantified the percentage of cells (*n* = 100) containing the integrated HHV-6 genome, confirming that the virus genome was indeed eliminated in about 70% of them (Fig. 3D). The 70% correspond to the percentage of Cas9-expressing cells, indicating that excision is very efficient provided that the Cas9 protein is present. To confirm this, we stained for Cas9 on the FISH slides. Excision occurred only in the presence of Cas9 and the 6A-gRNAs (Fig. 4, panel 4), while the viral genomes were readily detectable in the 30% of cells that did not express Cas9 (Fig. 4, panel 5) as well as in the controls.

To exclude the possibility that the virus reactivates upon excision, we assessed viral gene expression upon transfection of the gRNAs. RT-qPCR revealed that the essential immediate early gene U86 was not detectable (nd) after the excision over time (Fig. 3E). Taken together, our data show that the HHV-6A integrated genome can be successfully removed from chromosomes using the CRISPR/Cas9 system without inducing viral reactivation.

### Removal of the integrated HHV-6A genome by transient expression of Cas9.

Considering possible future clinical applications, we decided to assess the removal efficacy upon transient expression of Cas9 to avoid long-term expression of the protein in patient cells. We therefore cloned the PTG cassette into a Cas9 expression plasmid. This vector has the advantage of delivering the two components of the CRISPR/Cas9 system simultaneously, substantially shortening the selection process.

**FIG 5:**
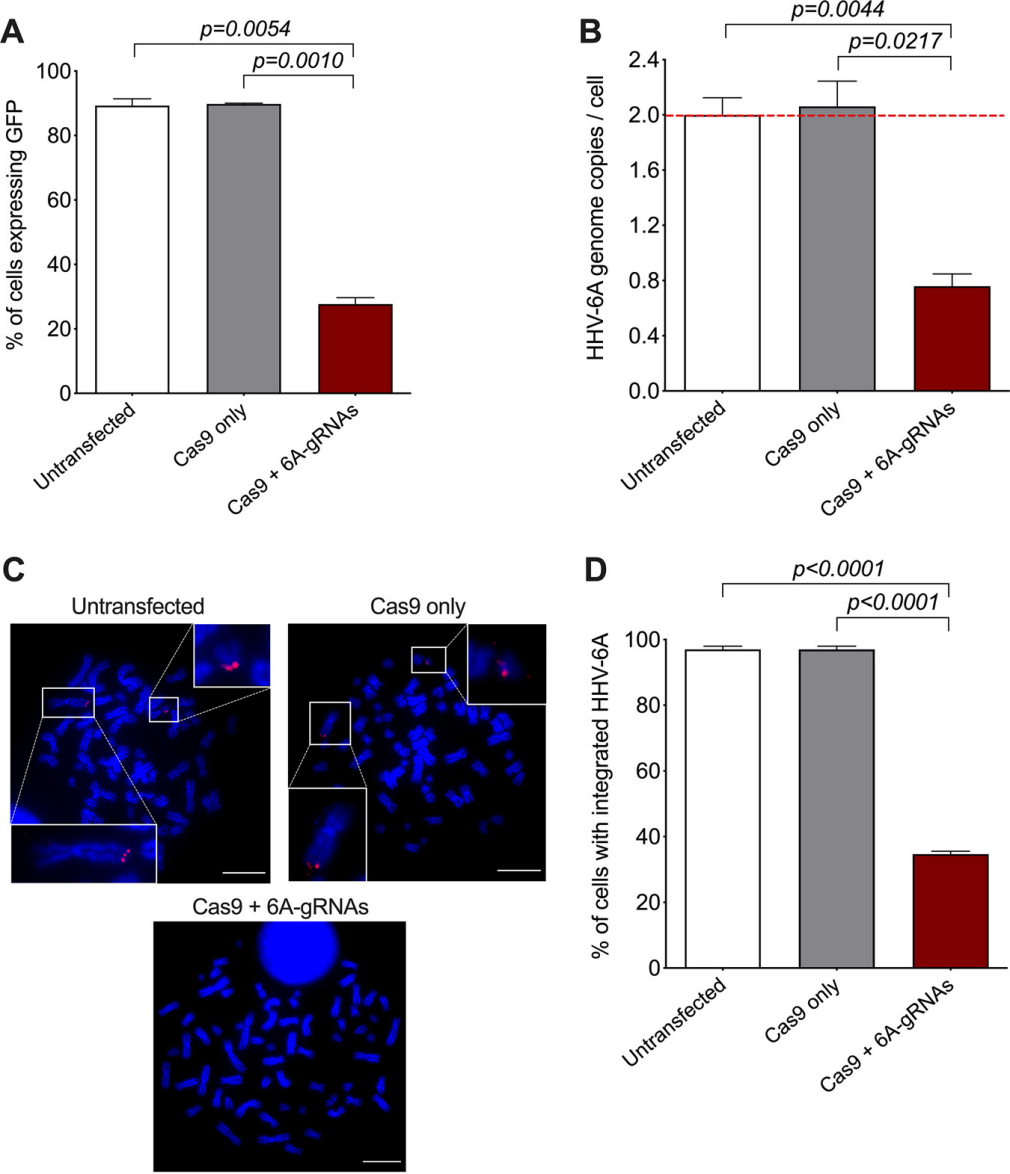
Removal of integrated HHV-6A genome via transient delivery of Cas9 + 6A-gRNAs. (A) Virus-encoded GFP expression was detected after TPA stimulation by flow cytometry in untransfected 293T-6A cells, cells transfected with Cas9 only, and cells transfected with Cas9 + 6A-gRNAs. Results are shown as means from three independent experiments (one-way ANOVA; *n* = 3; ±standard error of the mean). Representative FACS plots with gating are shown in Fig. S4 in the supplemental material. (B) Copy numbers of HHV-6A U86 gene were detected by qPCR. Results are shown as fold change (red dashed line) compared to untransfected 293T-6A cells, which harbor two copies of the integrated virus (one-way ANOVA; *n* = 3; ±standard error of the mean). (C) Integrated virus was detected by FISH using specific HHV-6A biotin-labeled probes and Cy3-streptavidin antibody (red). Metaphase chromosomes were stained with DAPI (blue). Representative images of untransfected 293T-6A cells, cells transfected with Cas9 only, and cells transfected with Cas9 + 6A-gRNAs are shown (bar, 10 μm). (D) One hundred interphase nuclei were examined for the presence or absence of the integrated virus (one-way ANOVA; *n* = 3; ±standard error of the mean).

The new plasmid and respective controls were delivered by transient transfection into 293T-6A cells, and the cells were selected with puromycin. As described above, the excision rate was assessed by FACS, qPCR, and FISH. Quantification of the GFP expression upon TPA stimulation revealed a significant reduction of the virus compared to the controls (Fig. 5A and Fig. S4). Quantification of viral genome copies by qPCR confirmed removal of the HHV-6A genome in about 65% of the transfected cells (Fig. 5B). Similarly, FISH images and their quantification revealed that the virus is no longer detectable in most cells transfected with the Cas9 + 6A-gRNAs (Fig. 5C and D). These data highlight that a transient delivery of Cas9 is a good alternative that facilitates the excision of the integrated HHV-6 genome without a prolonged expression of the Cas9 protein.

### Removal of integrated HHV-6A genomes from iciHHV-6A patient cell.

**FIG 6:**
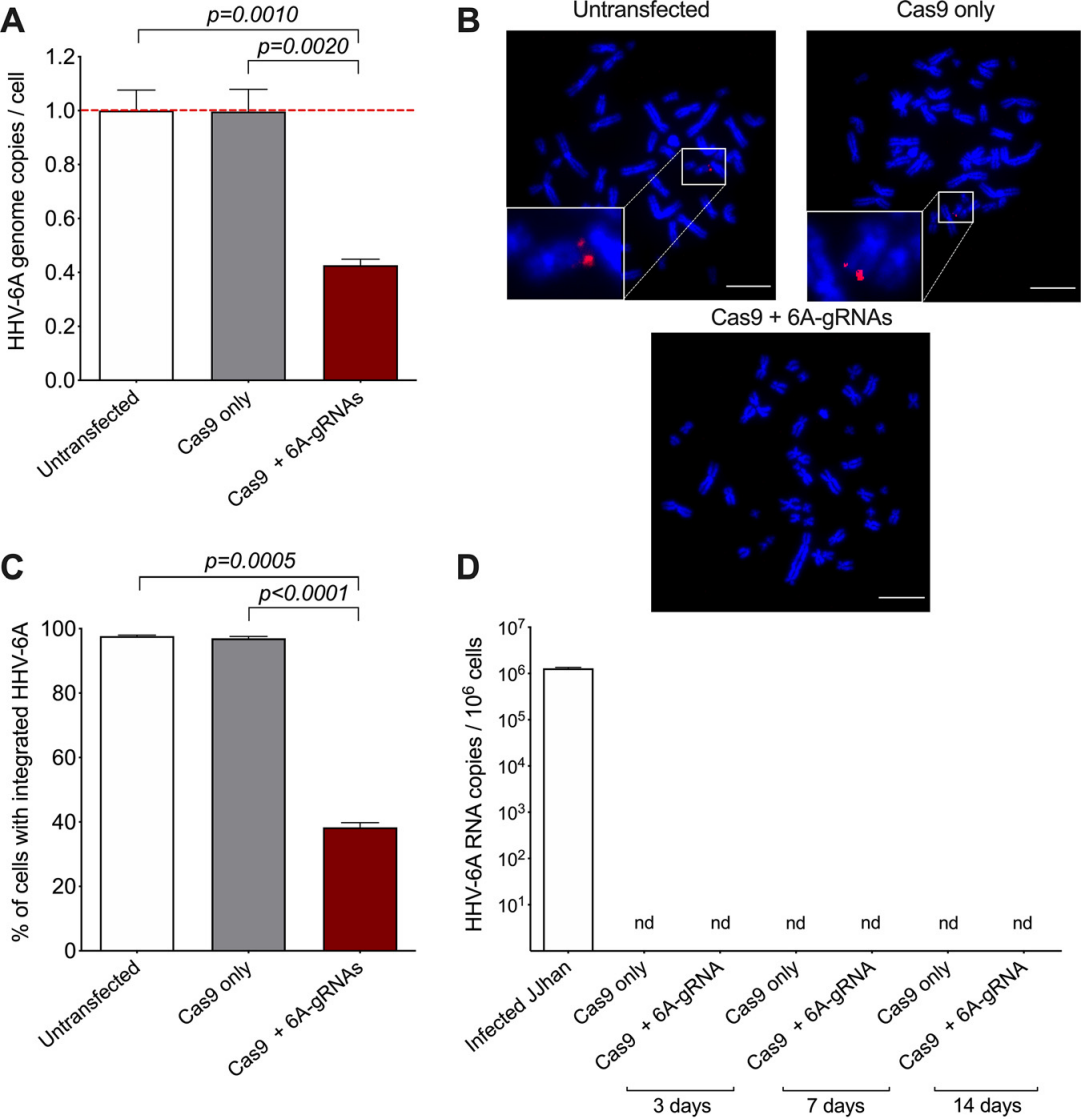
Removal of integrated HHV-6A genome from iciHHV-6A SMCs. (A) Copy numbers of the HHV-6A U86 gene were detected by qPCR. Results are shown as fold change (red dashed line) compared to untransfected iciHHV-6A SMCs, which harbor one copy of the integrated virus (one-way ANOVA; *n* = 6; ±standard error of the mean). (B) Integrated virus was detected by FISH as described for Fig. 5. Representative images of untransfected iciHHV-6 SMCs, cells transfected with Cas9 only, and cells transfected with Cas9 + 6A-gRNAs are shown (bar, 10 μm). (C) One hundred interphase nuclei were examined for the presence or absence of the integrated virus (one-way ANOVA; *n* = 3; ±standard error of the mean). (D) Total RNA was extracted at different time points after transfection. The absence of viral transcripts was confirmed by qPCR for the HHV-6 early gene U86 (*n* = 3; ±standard error of the mean). As a positive control, RNA from lytic infected JJhan cells was analyzed. nd, not detected.

To determine if the transient excision system also facilitates removal of the virus genome from iciHHV-6A patient cells, we used smooth muscle cells (SMCs). These cells have been immortalized by lentiviral delivery of the simian virus 40 (SV40) large T antigen and harbor 1 copy of the integrated iciHHV-6A genome, as reported in previous studies (54, 55). As described above, iciHHV-6A SMCs were transfected with the plasmid containing Cas9 + 6A-gRNAs and the cells were selected with puromycin. The excision efficiency was subsequently assessed by PCR and FISH. The qPCR data revealed a successful removal of the virus genome in about 57% of the transfected cells (Fig. 6A). These results were confirmed by FISH, showing a significant reduction of the number of cells harboring the integrated virus after the transfection with the Cas9 + 6A-gRNA plasmid (Fig. 6B and C). To exclude the possibility that the virus reactivates upon excision, we assessed viral gene expression upon delivery of the CRISPR/Cas9 system. RT-qPCR revealed that the immediate early gene U86 was not detectable after the excision over time (Fig. 6D).

Next, we determined if the presence of the remaining integrated virus genomes could be due to reintegration of the virus genome upon excision. Our previous studies demonstrated that the virus is integrated in chromosome 19 in these iciHHV-6A SMCs (55). In cells that still harbored the iciHHV-6A, the virus genome was still detected in chromosome 19, suggesting that the virus genome was not excised in those cells and did not integrate elsewhere upon excision (Fig. S3). Our data demonstrate that the virus genome can be removed from iciHHV-6 patient cells using a transient delivery of Cas9 + 6A-gRNAs.

### Removal of integrated HHV-6A genomes by sorting Cas9-expressing cells.

**FIG 7:**
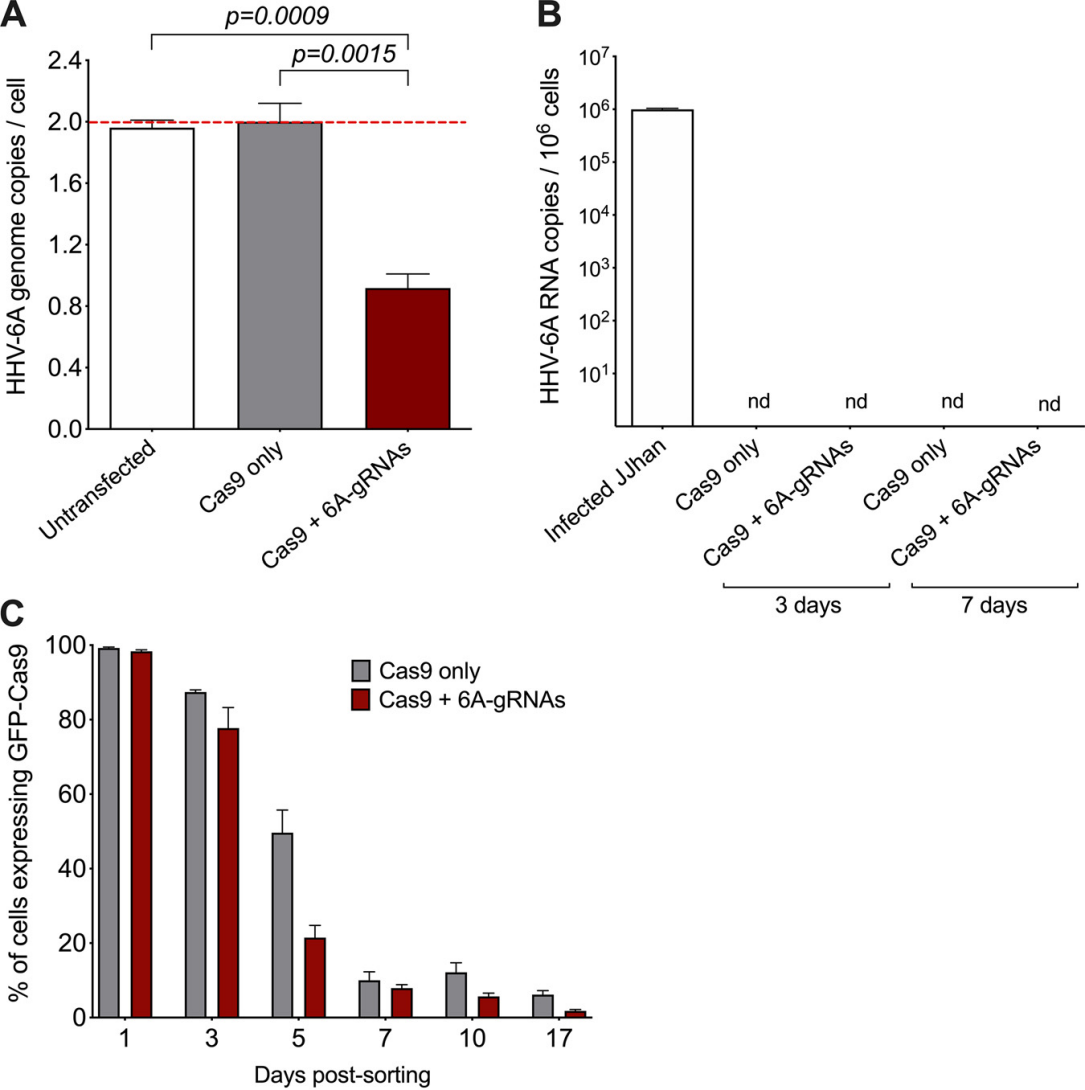
Removal of integrated HHV-6A genome in GFP-Cas9-sorted cells. (A) Copy numbers of the HHV-6A U86 gene were detected by qPCR. Results are shown as fold change (red dashed line) compared to untransfected 293T-6A cells, which harbor two copies of the integrated virus (one-way ANOVA; *n* = 3; ±standard error of the mean). (B) Total RNA was extracted at different time points after sorting. The absence of viral transcripts was confirmed by qPCR for the HHV-6 early gene U86 (*n* = 3; ±standard error of the mean). As a positive control, RNA from lytic infected JJhan cells was analyzed. nd, not detected. (C) Expression of the T2A-GFP-pCas9 was followed over time by flow cytometry (*n* = 3; ±standard error of the mean).

We demonstrated that the CRISPR/Cas9 system facilitates the removal of the integrated HHV-6A genome with both stable and transient Cas9 expression. However, a fraction of cells still retained the integrated virus, likely due to the lack of or low level of Cas9 expression. To overcome this problem, we used a GFP-tagged Cas9 protein to allow isolation of cells expressing a high level of the protein by FACS. We cloned the PTG cassette containing the 6A-gRNAs into a plasmid expressing Cas9-T2A-GFP and transfected the 293T-6A cells with the newly generated plasmid. As a control, we used the plasmid containing only Cas9-T2A-GFP. At 72 h after transfection, we sorted cells with high GFP expression, kept them in culture, and assessed the excision of HHV-6A by qPCR. Results revealed a 55% reduction of the viral genome copies compared to the controls (Fig. 7A). Surprisingly, this approach appeared to be less efficient than our previous approaches, despite the initially high Cas9 levels. To exclude the possibility that some of the detected genome copies account for a viral reactivation after excision and sorting, we performed RT-qPCR. However, no HHV-6A U86 transcripts were detected at different time points after sorting (Fig. 7B).

We therefore decided to track Cas9-T2A-GFP expression in the 293T-6A cells. This revealed a rapid loss of the Cas9 plasmid after sorting. Already after 5 days, the GFP expression was lost in most cells (Fig. 7C). These results suggest that despite the high Cas9 expression early on, the excision is less efficient, likely due to the rapid loss of the Cas9 expression.

### Consecutive removal of integrated HHV-6A genomes.

**FIG 8:**
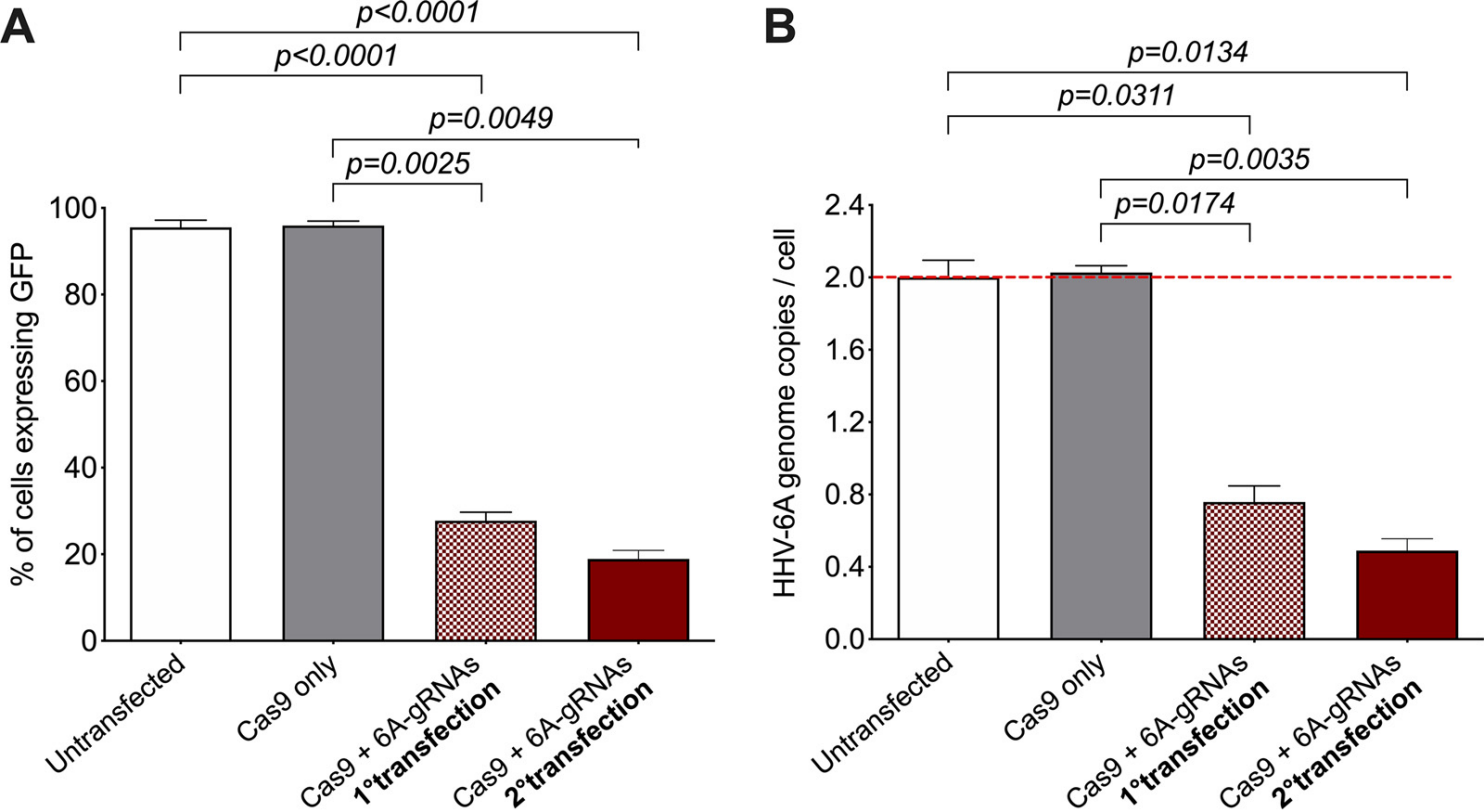
Removal of integrated HHV-6A genome through consecutive transfections. (A) Virus-associated GFP was detected by flow cytometry in untransfected 293T-6A cells, cells transfected with Cas9 only, cells transfected only once with Cas9 + 6A-gRNAs, and cells transfected twice with Cas9 + 6A-gRNAs. Results are shown as means from three independent experiments (one-way ANOVA; *n* = 3; ±standard error of the mean). (B) Copy numbers of HHV-6A U86 gene were detected by qPCR. Results are shown as fold change compared to untransfected HHV-6 293T cells, which harbor two copies of the integrated virus (one-way ANOVA; *n* = 3; ±standard error of the mean).

To further reduce the number of 293T-6A cells still harboring the integrated HHV-6A, we used two consecutive rounds of our transient delivery approach as described above. This aimed at reaching cells in which Cas9 was quickly downregulated or not expressed at all after the first transfection. The second delivery was performed 3 weeks later, when Cas9 expression of the first round had almost disappeared. Quantification of the GFP expression upon TPA stimulation revealed that a second round of the transient delivery eliminated the virus genome in more than 80% of the cells, although the additional reduction was not statistically significant (Fig. 8A). This further reduction was confirmed by the quantification of viral genomes by qPCR (Fig. 8B). These results highlight that two consecutive transient transfections of the CRISPR/Cas9 system can enhance the removal of the integrated HHV-6A genome.

### Analysis of the excision sites after the HHV-6A genome removal.

**FIG 9:**
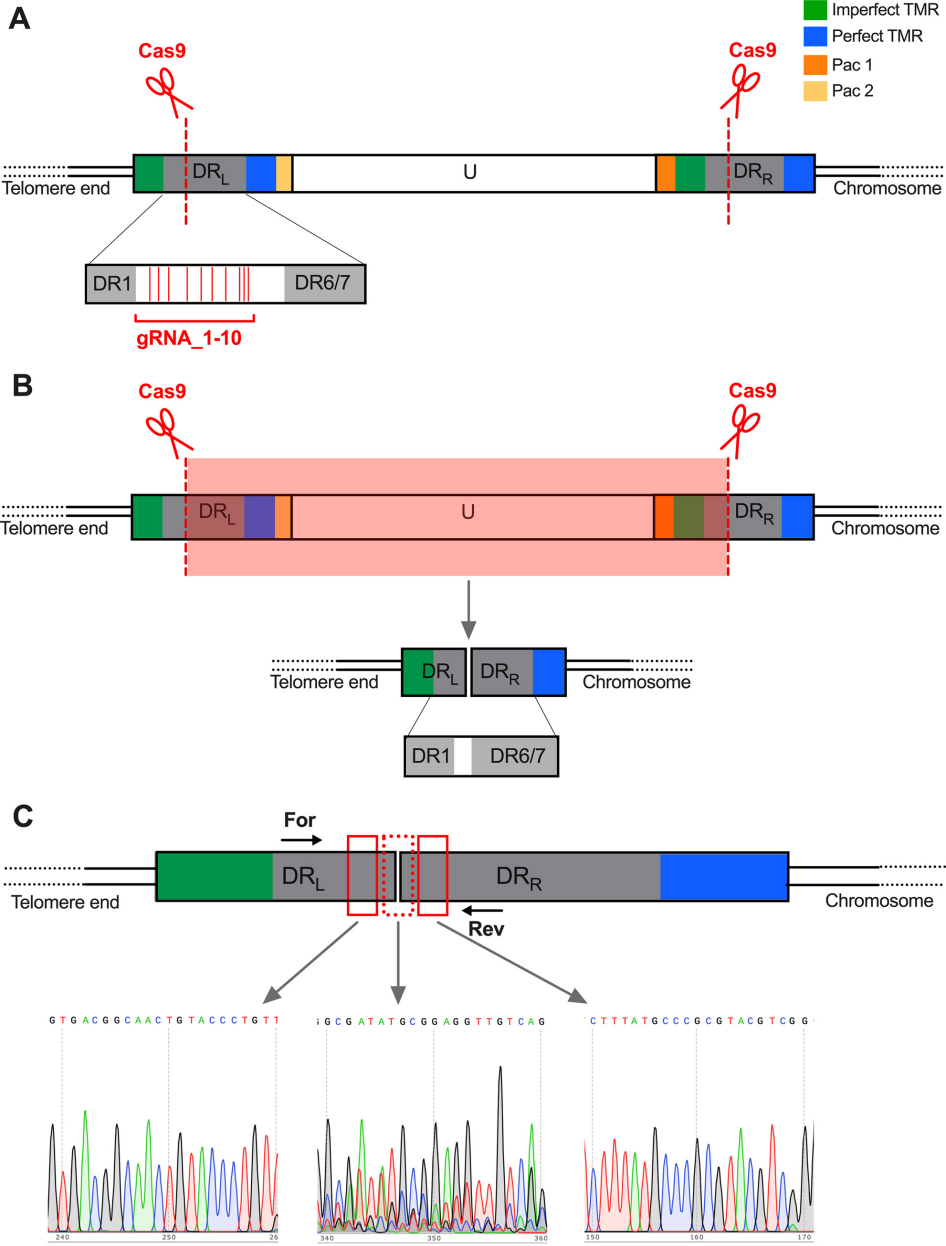
Excision site analysis after Cas9-based removal of the HHV-6A genome. (A) The set of 10 gRNAs targeted a noncoding region in the HHV-6A genome located between DR1 and DR6/7. (B) Following the Cas9 cut in both DRs, the U region, the Pac sequences, and part of the DRs are removed from the host genome. Non-homologous end-joining of the two resulting ends results in the generation of an incomplete DR harboring DR1 and DR6/7 from DR_L_ and DR_R_, respectively. (C) Specific primers were used to amply the excision site in the cell population after Cas9 treatment. The PCR product was the expected size and subsequently sequenced. Sequencing analyses reveal a homogeneous chromatogram profile in the area outside the excision site (left and right chromatograms). The area directedly targeted by the gRNA displays a more inhomogeneous profile, in line with error-prone nonhomologous end-joining in the polyclonal cell population (middle chromatogram).

Finally, we analyzed the excision sites in our cell populations after the HHV-6A genome removal. As described above, our set of HHV-6A gRNAs targeted the noncoding area in the DRs, between the DR1 and the DR6/7 locus (Fig. 2A and Fig. 9A). Following the successful Cas9-based excision, the full U region and part of the two DRs were removed from the host chromosome (Fig. 9B). After non-homologous end-joining of the two truncated ends, the newly formed junction retained only part of the two original DRs fused together in the host genome (Fig. 9B). To investigate the excision sites, we amplified and sequence the area targeted by the HHV-6 gRNAs (Fig. 9C). Sequencing results revealed the presence of the predicted junction with a high level of heterogeneity at the cut site due to the error-prone non-homologous end-joining mechanism (Fig. 9B). This indicated that most cuts occurred at the outermost target sites, highlighting the potential of reducing the number of gRNAs in future studies.

## DISCUSSION

In this study, we provide the first proof-of-concept that the integrated HHV-6A genome can be eliminated from the host telomeres using the CRISPR/Cas9 technology. Unlike most herpesviruses, which maintain their genome as circular episomes during latency, HHV-6A/B integrate into the telomere of human chromosomes. Integration makes it impossible to eradicate the virus with pharmacological drugs, which so far only moderately inhibit virus replication and do not prevent latency in the host (10–12).

Over the last several years, the CRISPR/Cas9 system proved to be a powerful tool for genome editing. Recent studies exploited this system to target the latent circular episomes of other herpesviruses, including herpes simplex virus 1 (HSV-1) and cytomegalovirus (CMV), aiming at disrupting single viral genes to abrogate lytic replication and prevent reactivation and/or episome maintenance (59–64). Since HHV-6A/B integrate their genome into the telomeres of latently infected cells, removal of the viral genome with its promoters, genes, and regulatory elements is certainly the best option. This approach could be also applied to other integrated (herpes)viruses.

Here, we designed HHV-6A-specific gRNAs targeting the direct repeats of the virus, to allow the release of the integrated viral genome from the host telomeres. The use of the polycistronic-tRNA-gRNA (Fig. 2A) allowed the delivery of multiple HHV-6-specific gRNAs from a single plasmid (56, 57). This approach is beneficial, as it would allow the removal of a broad range of viral strains without the need for adapting the system to each specific strain. In our initial experiments, we constitutively expressed Cas9 by lentivirus delivery. Upon expression of HHV-6A-specific gRNAs, the virus genome was removed in most cells, providing the first evidence that our system is functional (Fig. 3). Although we observed that the integrated Cas9 gene was slowly downregulated over time, its long-term presence in the cells, and perhaps reemerging expression, could result in an unspecific off-target Cas9 activity. To avoid this, we decided to establish a system that allows transient expression of Cas9 and showed that its expression is lost over time. Our data show how HHV-6A excision can be achieved without constitutive Cas9 expression in both latently infected cells generated *in vitro* and iciHHV-6A patient cells. The transient Cas9 expression makes the system more appealing for future possible application.

During the establishment of latency, herpesviruses silence most of their genes to escape the host immune response and persist mostly undetected by the host immune system. This is also true for HHV-6, as its integrated genome is silenced in a compact state and contains a repressive chromatin profile (54). Until now, only a few HHV-6 transcripts have been detected during latency (65–67). The U94 protein has been detected at higher levels during latency than during lytic infection, and its expression has been associated with inhibition of cell migration and increased immune response (68–71). Latent HHV-6 gene expression could therefore influence host cells and the health of the host not only during virus reactivation but also during the latent phase. Supporting an active role of the integrated HHV-6 genome, recent studies highlighted a tissue-specific RNA expression of some viral genes in iciHHV-6 individuals, indicating that certain genes are expressed at some point in time even without complete viral reactivation (65, 66). For instance, specific viral transcripts have been detected in the brain of iciHHV-6A individuals (65), consistent with several studies that associate HHV-6A with various neurological diseases, including multiple sclerosis and Alzheimer’s disease (72–78).

It has been hypothesized that the first step of HHV-6A/B reactivation is the release of the virus genome from the chromosomes through a t-loop formation. Recombination between the host telomere and the viral TMR within the DRs would result in the release of a replication-competent circular viral genome with one full-length DR (79–81). Telomere shortening in most somatic cells over time could also play a role in the release and reactivation of the virus, an aspect that should be investigated in the future. Interestingly, our data revealed that the release of the HHV-6A genome after excision does not lead to viral reactivation, as expression of the immediate early U86 gene was not detected in our experiments. These data suggest that the simple viral genome release is *per se* not enough to trigger HHV-6A reactivation. Indeed, the excised virus genome lacks parts of its DRs, retained on the host genome, and does not harbor TMRs at its ends. Therefore, the excised sequence likely will not be able to reconstitute a functional circular viral genome capable of initiating virus replication. On the other hand, the specific circumstances and/or the trigger behind complete viral reactivation is still not fully understood.

Furthermore, our data showed that no reintegration can be detected after the excision of the HHV-6A genome from the host telomeres (see Fig. S3 in the supplemental material). It appears that the mere presence of the excised quiescent HHV-6A genome is not enough to integrate once again. Importantly, following excision with our gRNAs, the released viral genome would lack its TMRs at the end of the virus genome (Fig. 2A). As the viral TMRs have been shown to be required for efficient virus integration, a reintegration event would be rather unlikely (29).

Upon the Cas9-based cleavage of the HHV-6A genome, the U region and large parts of the DRs would be removed. Subsequently, the remaining DR_L_ and DR_R_ are fused together by non-homologous end-joining as observed in our PCR analyses (Fig. 9) and retained in the host telomere. The presence of a partial DR without its Pac sequences is also observed upon the release of the virus genome during reactivation via the proposed t-loop model (79, 81). Compared to the unique region, the two DRs remain poorly characterized. Interestingly, DR1 and DR6 loci have been shown to be dispensable for virus replication *in vitro*, as their deletion does not impair virus propagation in T-cell lines (82). As HHV-6A/B specifically integrate into the host telomeres, the excision of the virus genome could affect telomere length. Our sequencing data revealed that the region adjacent to the excision sites remained intact in the cell population, suggesting that nonhomologous end-joining successfully fuses the two ends while maintaining the original telomere present at the DR_R_. In addition, telomere lengthening might also occur in the cells used in this study as they are telomerase positive. Intriguingly, a recent study revealed the presence of long telomere sequences (from 5.0 to 13.3 kbp) between the subtelomeres and the virus genome using a whole-genome optical mapping technology. This implies that HHV-6A/B integration occurs at or near the end of the telomere rather than in proximity to the subtelomeres as previously thought (33). In turn, this suggests that even if no nonhomologous end-joining took place and the DRs eroded, there would still be long telomeres present at the new end of the chromosome.

Taken together, our results highlight that the virus can be successfully eliminated from the telomeres using specific gRNAs against terminal regions of the HHV-6A genome, without triggering viral reactivation. In addition, we showed how a transient Cas9 expression is sufficient to achieve viral excision, without the need of a long-lasting Cas9 expression. Our study provides the first proof-of-concept that the integrated HHV-6A genome can be successfully removed from iciHHV-6 patient cells and latently infected cells generated *in vitro*, providing the basis for future clinical applications. As iciHHV-6 patients harbor the integrated viral genome in every cell of the body, a complete clearance of the virus from these individuals remains impossible. However, our approach could be used to eradicate the virus from specific cell populations or from a subset of cells. For example, this method could be applied to cells used in hematopoietic stem cell transplantations, to clear them of the integrated virus and avoid virus reactivation that can lead to encephalitis, graft rejection, and graft-versus-host disease (GvHD) in immunocompromised patients. Beyond the potential clinical applications, this approach could provide important insights into HHV-6 biology. For example, removal of the virus genome from cells of iciHHV-6 individuals would reveal how the integrated virus genome with its promoters and regulatory elements influences the host cell. This would be a crucial step toward understanding the influence of this virus present in every cell of millions of people.

## MATERIALS AND METHODS

### Cell lines and virus.

HHV-6A-latently infected 293T (293T-6A) cells harboring two copies of the integrated virus genome (strain U1102) were previously generated and described (54, 55). Cells were cultured in Dulbecco’s modified Eagle’s medium (DMEM) supplemented with 10% fetal bovine serum (FBS; Pan Biotech), 1% penicillin-streptomycin, and 5 μg/mL Plasmocin (InvivoGen, San Diego, CA, USA). Human smooth muscle cells (SMCs) harboring the integrated iciHHV-6A genome were described previously (54). SMCs were cultured in M199 medium (Pan Biotech) supplemented with 20% FBS (Pan Biotech), 1% penicillin-streptomycin, and 5 μg/mL Plasmocin (InvivoGen, San Diego, CA, USA). All cells were cultured in a humidified incubator with 5% CO_2_.

### Lentivirus delivery of Cas9.

Cas9 was constitutively expressed upon lentivirus delivery as described previously (83, 84). Briefly, Cas9 lentiviruses were produced by cotransfecting 293T cells with the two packaging vectors PCMV-VSV-G and pCMVDR8.91 and the transfer vector pSicoR-CRISPR-PuroR expressing the S. pyogenes Cas9 gene (kindly provided by Robert Jan Lebbink, University Medical Center Utrecht) (60, 85). Lentiviruses were harvested at 48 h post transfection and delivered as described previously (83, 84). Transduction of 293T-6A cells was performed by spin inoculation at 1,200 × *g* and room temperature for 2 h. After transduction, cells were selected using 1 μg/mL of puromycin (Invitrogen, Waltham, MA, USA) for 5 days.

### Generation of HHV-6A-specific gRNAs.

HHV-6A-specific gRNAs (6A-gRNAs) were identified using the GPP sgRNA designer (Broad Institute, https://portals.broadinstitute.org/gpp/public/analysis-tools/sgrna-design). The gRNAs with the highest specificity for HHV-6A direct repeat (DR) regions were selected. The gRNAs were directed against the noncoding region of the direct repeats, between DR1 and DR6 (Table 1, gRNA sequences, and Fig. 1A). As a control, we used gRNAs specific for the herpesvirus Marek’s disease virus (MDV) published previously (58).

To deliver 10 different HHV-6 gRNAs, we used a polycistronic-tRNA-gRNA (PTG) system to produce multiple functional gRNAs from a single U6 promoter as described previously (56, 57). Briefly, each individual gRNA was interspersed with tRNA sequences (Fig. 2B). The gRNA/tRNA cassette was transcribed, and the pre-tRNA sequences were subsequently precisely cleaved at both ends by the endogenous RNases, releasing the 10 individual HHV-6A gRNAs (86–89) (Fig. 2B). The designed U6-PTG.sg6A cassette, harboring the 6A-gRNAs and the tRNA sequences under the control of the U6 promoter, was synthesized by GeneArt Gene Service (BioPark, Regensburg, Germany).

### Generation and delivery of gRNAs and Cas9 + gRNA vectors.

The U6-PTG.sg6A cassette was first cloned into the PLKO5.sgRNA.EFS.PAC vector (Addgene catalog no. 57825) (90) containing a hygromycin selection cassette instead of puromycin as described previously (58). The newly generated plasmid PLKO5-PTG.sg6A was used to deliver the 6A-gRNAs into the 293T-6A-Cas9 cells. Transfection was performed using the polycation polyethylenimine (PEI), and transfected cells were selected using 400 μg/mL hygromycin for 7 days.

To generate a plasmid that expresses both Cas9 and the 6A-gRNAs, the U6-PTG.sg6A cassette of the PLKO5.PTG.sg6A plasmid was cloned into the pSicoR-CRISPR-PuroR Cas9 plasmid. First, EcoRI and PpuMI sites were used to insert two new BsrGI restriction sites at the extremity of the U6-PTG.sg6A cassette using a complementary oligonucleotide annealing approach (oligonucleotides used are shown in Table 2). The cassette was then cloned into the pSicoR-CRISPR-PuroR Cas9 plasmid using the new BsrGI restriction sites. Correct insertion and orientation of the cassette were confirmed by restriction fragment length polymorphism (RFLP) and Sanger sequencing (LGC Genomics, Berlin, Germany). The new final plasmid pCas9-PTG.sg6A was delivered into 293T-6A cells and iciHHV-6A SMCs. Transfection of 293T-6A cells was performed using PEI, while iciHHV-6A SMCs were transfected using X-fect single shots (TaKaRa Bio, Kusatsu, Japan) according to the manufacturer’s guidelines. Cells were selected using 1 μg/mL of puromycin (Invitrogen, Waltham, MA, USA) for 5 days.

**TABLE 2:**
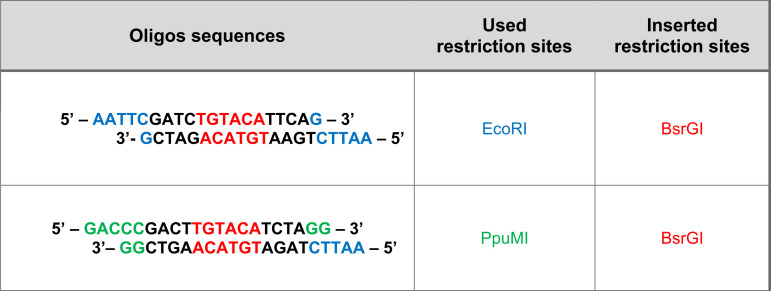
Annealed oligonucleotides for the insertion of BsrGI sites into the U6-PTG.sg6A cassette

To visualize and sort Cas9-expressing cells, we cloned the U6-PTG.sg6A cassette into the vector pSpCas9(BB)-2A-GFP (Addgene catalog no. 48138), containing GFP fused to Cas9 via a T2A ribosome skipping motif (91) using BsrGI. Correct insertion and orientation of the cassette were confirmed by RFLP and Sanger sequencing (LGC Genomics, Berlin, Germany). Transfection of the new plasmid pCas9.GFP-PTG.sg6A into 293T-6A cells was performed using PEI. At 72 h after transfection, cells expressing high levels of Cas9-T2A_GFP were sorted using a FACSAria III cell sorter (BD Biosciences).

### Cas9 protein detection by flow cytometry and immunofluorescence.

To determine which cells express Cas9, we stained and analyzed them by FACS. Cells were fixed with 4% paraformaldehyde (PFA) (Sigma-Aldrich, St. Louis, MO, USA) for 30 min. Permeabilization was achieved with 0.3% Triton X-100 (Merck, Darmstadt, Germany) in phosphate-buffered saline (PBS) for 10 min, and blocking was performed with 10% bovine serum albumin (BSA) (AppliChem, Darmstadt, Germany) plus 0.3% Triton X-100 in PBS for 45 min. Cas9 was detected using the mouse anti-Cas9 monoclonal antibody (MAb) (7A9-3A3) conjugated with Alexa Fluor 647 (catalog no. 48796; Cell Signaling, Danvers, MA, USA) at a 1:200 dilution in PBS. The Cas9-Alexa 647 signal was detected and quantified using a CytoFlex S instrument (Beckman Coulter, Brea, CA, USA). Results were analyzed using the FlowJo software.

In addition, Cas9 protein expression was detected by immunofluorescence. Cells were seeded on precoated slides in 24-well plates. Cells were fixed, permeabilized, and blocked as described above for the FACS staining. Cas9 was detected by using the anti-d-Tag mouse MAb (ABM, Richmond, BC, Canada) at a 1:250 dilution in PBS to detect the synthetic peptide DYKDDDDK in the Cas9 protein. The secondary Alexa Fluor 568-conjugated anti-mouse antibody (Invitrogen, Waltham, MA, USA) was used at a 1:2,000 dilution in PBS. Images were acquired with a Zeiss M1 microscope using a 100× objective and the AxioVision software (Carl Zeiss, Inc.). Images were analyzed using ImageJ (https://imagej.nih.gov/ij/) and its specific processing package Fiji (https://imagej.net/Fiji).

### Detection of the virus-encoded GFP upon stimulation.

293T-6A cells were stimulated with phorbol 12-myristate 13-acetate (TPA; Sigma-Aldrich) (1 μg/mL) for 24 h to induce the expression of the GFP under the control of the HCMV major intermediate early promoter as described previously (54). The GFP signal was detected and quantified by flow cytometry using a CytoFlex S Instrument (Beckman Coulter, Brea, CA, USA). Results were analyzed using the FlowJo software.

### Quantification of the HHV-6 genome by qPCR.

DNA was isolated from samples using the Zymos Quick-DNA viral kit following the manufacturer’s instructions. qPCR was performed as described previously using primers and probes specific for the HHV-6A U86 and the cellular β_2_M gene using the SensiFAST master mix (Bioline, Memphis, TN, USA) (29, 35). The viral genome copies (U86) were subsequently normalized against the cellular β_2_M gene. The qPCRs were performed using an ABI 7500 Fast system (Applied Biosystems, Waltham, MA, USA) (Fig. 3 and 5) or a qTOWER^3^ G qPCR (Analytik Jena, Jena, Germany) (Fig. 6, 7, and 8).

### Detection of HHV-6A immediate early gene expression.

To assess HHV-6A reactivation, we assessed the expression of an immediate early gene (U86) that is expressed during virus replication and reactivation. Total RNA of 293T-6A and iciHHV-6A SMCs upon virus genome excision was extracted using the RNeasy Plus minikit (Qiagen, Hilden, Germany) according to the manufacturer’s instructions. Potential carryover of DNA contamination was removed using the RQ1 DNase (Promega, Madison, WI, USA) according to the manufacturer’s instructions. cDNA was generated using the high-capacity cDNA reverse transcription kit (Applied Biosystems, Waltham, MA, USA). qPCR was performed as described above.

### Detection of HHV-6A genomes by FISH.

The integrated HHV-6A genome was detected by fluorescence *in situ* hybridization (FISH) as described previously (29, 92, 93) with the following modifications. Briefly, HHV-6A probes were generated using the HHV-6A bacterial artificial chromosome (BAC) (U1102 strain) and labeled using High-Prime biotin (Sigma-Aldrich, St. Louis, MO, USA). Detection of the HHV-6A probe signal was achieved using Cy3-streptavidin (1:1,000; Roche, Basel, Switzerland). A chromosome 19-specific probe was generated using High-Prime digoxigenin (DIG) (Sigma-Aldrich, St. Louis, MO, USA) and a chromosome-specific BAC clone (RPCI-11; Source BioScience, Nottingham, England) as described previously (93). Detection of chromosome probe signal was achieved using anti-DIG fluorescein isothiocyanate (FITC) Fab fragments (1:200; Roche, Basel, Switzerland). To obtain an adequate number of metaphases, 293T-6A cells were treated with colcemid for 5 h, while iciHHV-6 SMCs were treated with colcemid for 16 h prior to sample preparation.

To detect Cas9 in HHV-6A FISH samples, staining of the Cas9 protein was performed with the mouse anti-Cas9 MAb (7A9-3A3) conjugated with Alexa Fluor 647 (1:200; catalog no. 48796; Cell Signaling, Danvers, MA, USA). Images were acquired with a Zeiss M1 microscope using a 100× objective and AxioVision software (Carl Zeiss, Inc.). Images were analyzed using ImageJ (https://imagej.nih.gov/ij/) and its specific processing package Fiji (https://imagej.net/Fiji).

### Sequencing of the excision junction after HHV-6 genome removal.

DNA was isolated from samples using the Zymos Quick-DNA viral kit following the manufacturer’s instructions. The primers listed in Table 3, located outside the 6A-gRNA target sequences, were used to amplify the region of the excision junction. PCR amplification was performed using the PrimeSTAR GXL DNA polymerase (TaKaRa Bio, Kusatsu, Japan) following the manufacturer’s instructions. The PCR products were purified using the high-yield PCR/gel extraction kit (Süd-Laborbedarf GmbH, Gauting, Germany). The purified PCR products were sequenced by Sanger sequencing (LGC Genomics, Berlin, Germany), and results were analyzed using SnapGene software (https://www.snapgene.com/). The HHV-6A strain U1102 genome was used as a reference genome for the analysis.

**TABLE 3:** Primers for the amplification of the excision junction region

Primer	Sequence
Forward	5′-AAAGAGTCACGTCCGTTCC-3′
Reverse	5′-ACGTCACGTGGAAAAATGTTT-3′

### Statistical analysis.

All statistical analyses were performed using GraphPad Prism. One-way analysis of variance (ANOVA) was performed, with Geisser-Greenhouse correction and Tukey’s multiple-comparison test.

### Ethics approval and consent to participate.

Ethics approval and consent to participate are not applicable.

### Data availability.

The data sets supporting the conclusions of this article are included within the article and its supplemental material.
