# Supplementary material for: Excision of Integrated Human Herpesvirus 6A Genomes Using CRISPR/Cas9 Technology
Source: Microbiol Spectr. 2023 Mar 16;11(2):e00764-23. doi: 10.1128/spectrum.00764-23 (PMC10100985; doi:10.1128/spectrum.00764-23)
Supplement: Supplemental file 1 — Fig. S1 to S4. Download spectrum.00764-23-s0001.pdf, PDF file, 0.6 MB [file spectrum.00764-23-s0001.pdf]

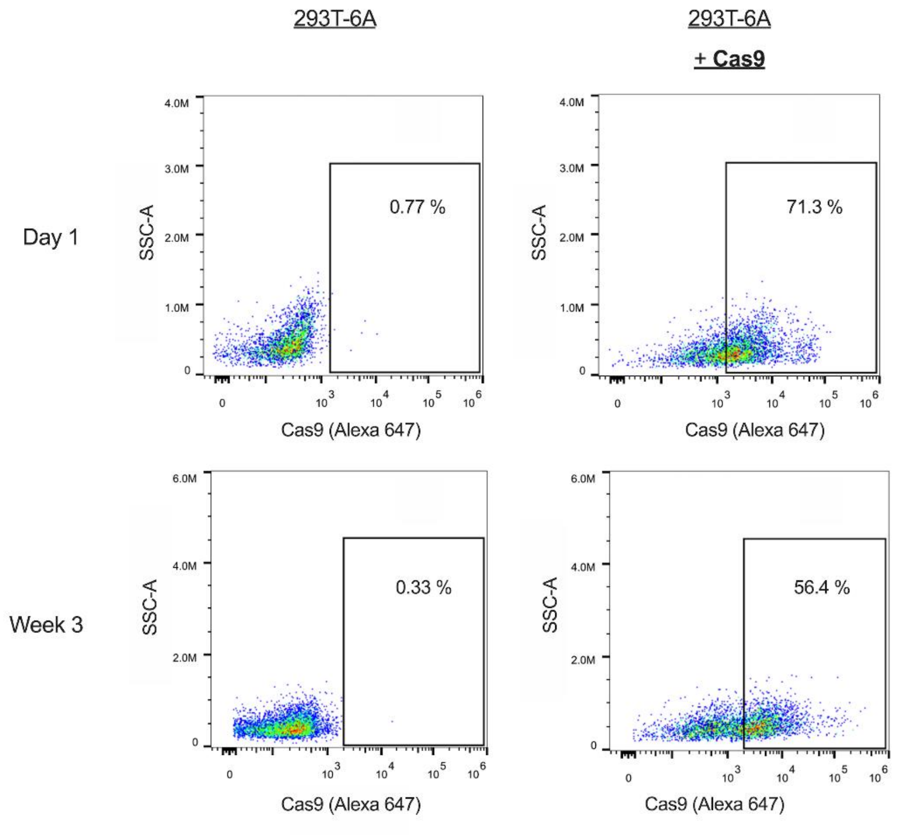

Figure S1- Quantification of Cas9 expression by flow cytometry

Representative FACS plots of Cas9 expression in Cas9 transduced 293T-6A (mean of multiple experiments shown in Figure 1A). Negative gating was set on Untransduced 293T-6A. Cas9 staining was performed with Cas9 (7A9-3A3) Alexa Fluor 647-conjugated antibody.

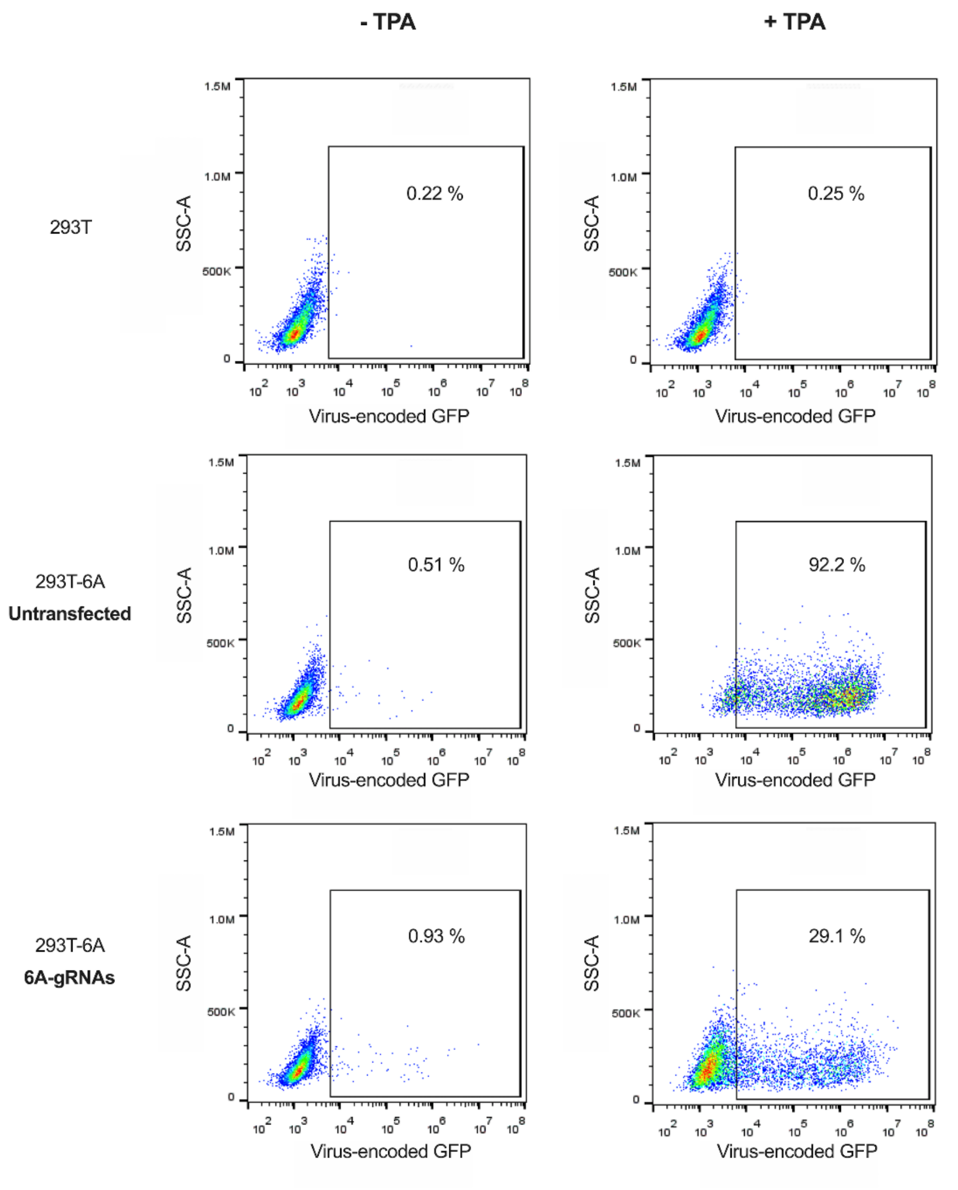

Figure S2 - Quantification of virus-associated GFP by FACS in Cas9 transduced cells

Representative FACS plots of virus-associated GFP expression, w/o TPA stimulation, in 293T-6A-Cas9 cells (mean of multiple experiments shown in Figure 3A). Negative gating was set on uninfected 293T cells stimulated with TPA.

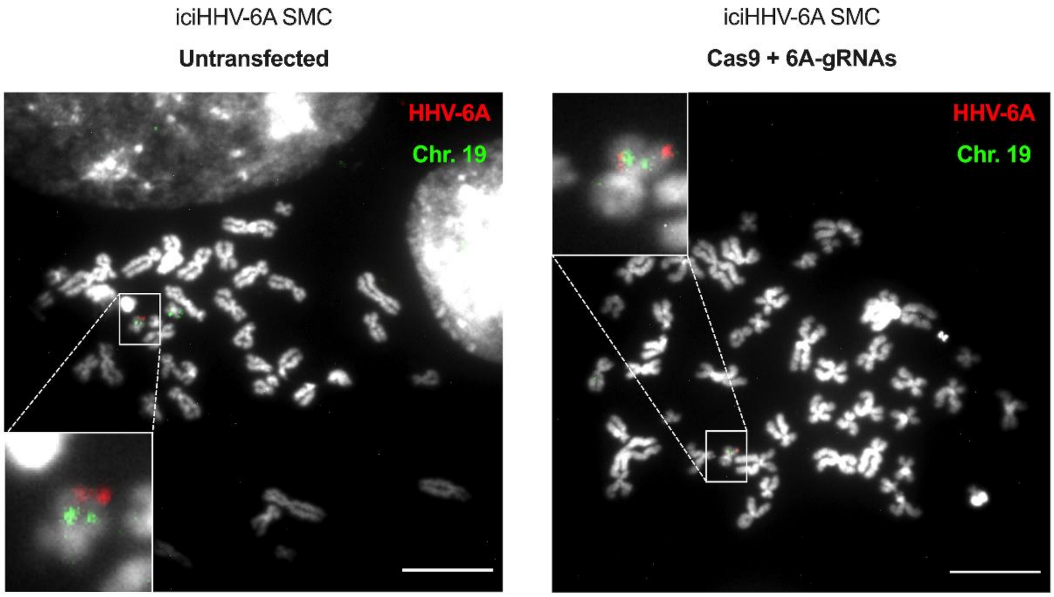

Figure S3 – identification of chromosome harboring integrated HHV-6A before and after removal

Integrated virus was detected by FISH using specific HHV-6A biotin-labeled probes and Cy3-Streptavidin antibody (red). Chromosome 19 was detected using specific DIG-labeled chromosome probe and anti-DIG FITC fragments [85]. Representative images of untransfected iciHHV-6 SMCs and transfected with Cas9 + 6A-gRNAs are shown (Scale bar 10µm).

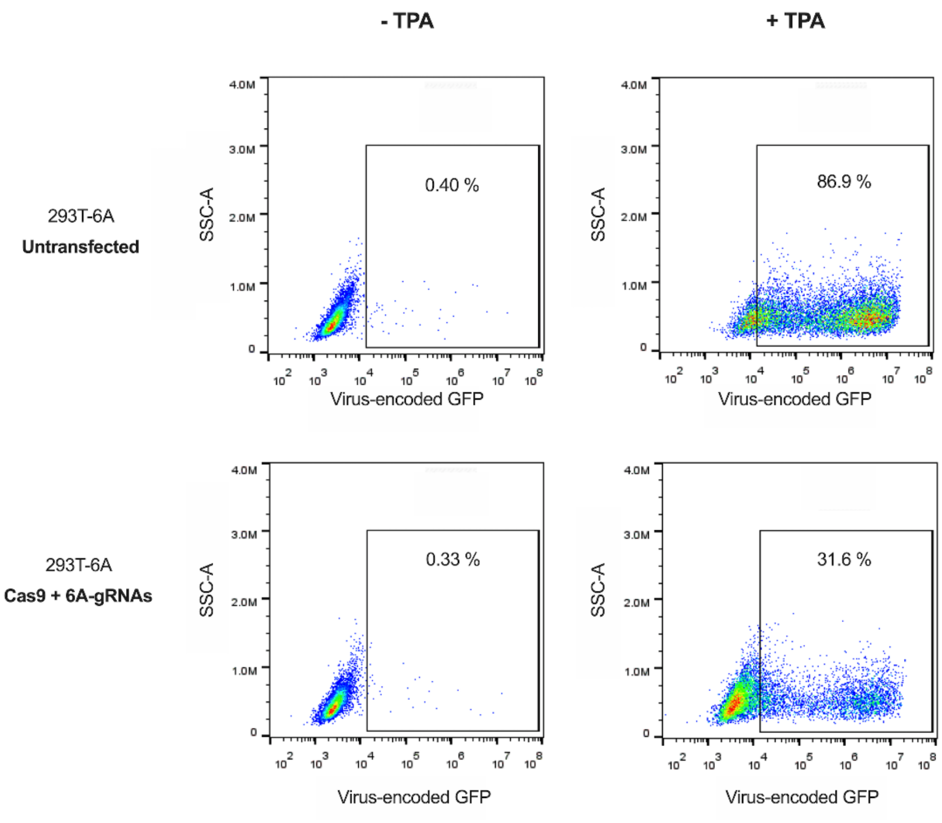

Figure S4 - Quantification of virus-associated GFP by FACS after transient transfection of Cas9 + 6A-gRNAs

Representative FACS plots of virus-associated GFP expression, w/o TPA stimulation, in 293T-6A transfected with Cas9 + 6A-gRNAs (mean of multiple experiments shown in Figure 5A). Negative gating was set on uninfected 293T cells stimulated with TPA.
